# Quantitative analysis of the relationship between maxillary incisors and the incisive canal by cone-beam computed tomography in an adult Japanese population

**DOI:** 10.1186/s40510-017-0181-1

**Published:** 2017-08-14

**Authors:** Tomonari Matsumura, Yuji Ishida, Ayako Kawabe, Takashi Ono

**Affiliations:** Orthodontic Science, Department of Oral Health Sciences, Graduate School of Medical and Dental Sciences, Tokyo Medical and Dental University, 1-5-45 Yushima, Bunkyo-ku, Tokyo, 113-8549 Japan

## Abstract

**Background:**

In setting goals for orthodontic treatment, determining the morphologies of the alveolar bone and maxillary incisor root is important for avoiding root resorption, dehiscence, and fenestration. This study aimed to analyze the configurational relationships among maxillary incisors, the alveolar border, and the incisive canal by cone-beam computed tomography (CBCT).

**Methods:**

Cone-beam CT images of 93 orthodontic patients were evaluated for length of the incisive canal (L); angles between the palatal plane and the maxillary alveolar border (θ1), the incisive canal (θ2), and maxillary incisor (θ3); distance from the left maxillary incisor to the incisive canal (D); and cross-sectional areas of the incisive canal (CSAs) at three vertical levels. Comparison of variables between male and female patients was performed with the two-sample *t* test. Correlations between parameters were examined by Pearson’s correlation analysis and Bonferroni correction for multiple comparisons.

**Results:**

Male patients exhibited significantly greater values of L than female patients. There were significant positive correlations between θ1 and θ2, θ2 and θ3, and θ3 and θ1. While the value of D was the lowest at the oral opening, that of the cross-sectional area of the incisive canal (CSA) was the greatest at the incisal root apex.

**Conclusions:**

This study demonstrated that the incisive canal had large inter-individual variability, and the proximity between the incisive canal and the incisal root could not be precisely predicted by the conventional cephalograms. Therefore, pre-treatment CBCT examination should be recommended when a large amount of maxillary anterior retraction and/or intrusion is planned in orthodontic diagnosis.

## Background

Orthodontic patients commonly require improvement of facial esthetics. Spatial position of the maxillary incisor is a critical factor in both facial esthetics and maxillofacial function [1]. Therefore, three-dimensional (3D) position and inclination of the maxillary incisor is regarded as a dominant factor when setting goals for orthodontic treatment [1–5]. With regard to maxillary incisor movement, Ackerman et al. presented the concept of “envelope of discrepancy” [6], which describes the limitations of the range of orthodontic movement of the maxillary incisor [7]. Contact with hard tissue structures, such as the labial, palatal, or incisive canal cortical plates, is a risk factor for apical root resorption in the maxillary incisor [8–10], and it is one of the iatrogenic complications of orthodontic treatment. In addition, excessive tooth movement during orthodontic treatment can induce root deviation from the alveolar housing of dentition, leading to dehiscence and fenestration [11]. Limitation of maxillary incisor movement in conjunction with cephalometric analysis has been proposed in order to avoid these potential complications; however, this approach remains controversial [12, 13].

In orthodontics, diagnosis and treatment planning have generally been performed by two-dimensional (2D) analysis of lateral and anteroposterior cephalometric measurements. However, the incisive canal and cortical plate on the sagittal plane of maxillary incisors cannot be precisely evaluated in conventional cephalometric radiographs. Recent developments in 3D analysis of dental cone-beam computed tomography (CBCT) images could help obtain more detailed information. Several studies using dental CBCT have suggested that determining the morphologies of maxillary incisor roots, the incisive canal, and the maxillary alveolar bone is important when setting goals for orthodontic treatment [8–10, 14, 15].

While previous CT and CBCT studies have clarified the anatomical relationships between maxillary incisors and the incisive canal [16], they suffered from inadequate image resolution for precise evaluation of alveolar bone shape and thickness. The aim of this study was to analyze the configurational relationships between maxillary incisors and the incisive canal in the anterior region of the maxillary alveolar bone using CBCT images.

## Methods

### Subjects

From among patients between the ages of 18 and 39 years who sought orthodontic treatment at the Tokyo Medical and Dental University Dental Hospital between 2012 and 2015, only those who required CBCT for diagnosis and treatment planning were selected. Written consent was obtained from all subjects after explanation of the research aims and goals.

The exclusion criteria were (1) history of orthodontic treatment, (2) missing or supernumerary maxillary incisors, 3) midline deviation of maxillary incisors ≥2 mm from the facial midline, (4) prosthodontic treatment of maxillary incisors, 5) evident nasopalatine pathology (e.g., nasopalatine duct cysts), (6) history of trauma to maxillary incisors, and 7) congenital anomalies (e.g., cleft lip and palate). Based on the inclusion and exclusion criteria, 93 subjects (male, 31; female, 62; mean age, 24.3 ± 5.6 years) were finally selected. Their skeletal pattern was Class I, Class II, and Class III and their mean ANB was 3.1 ± 3.5 (range −4.6–9.0).

### CBCT

**Fig. 1:**
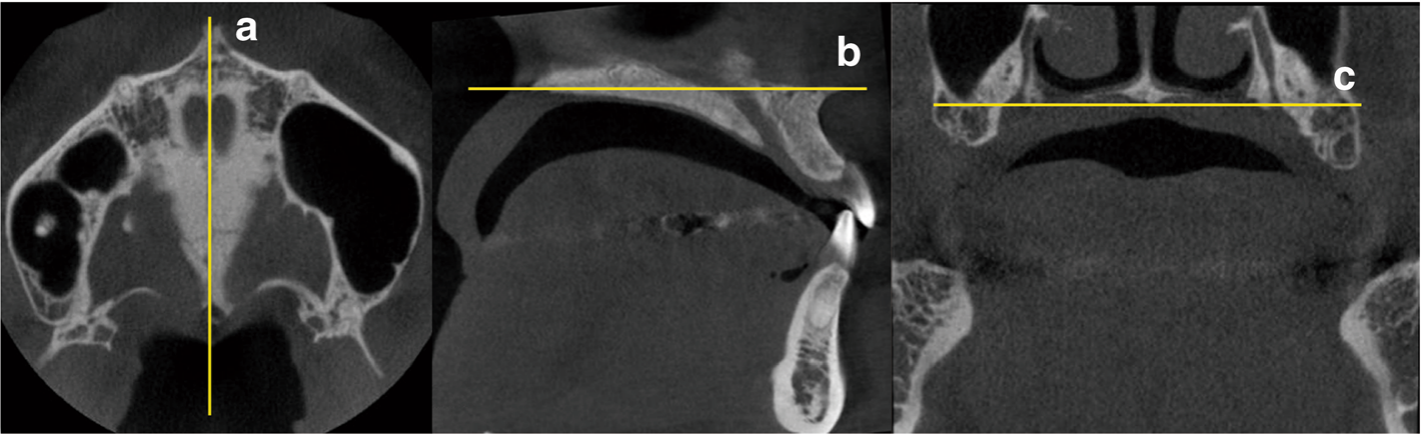
*Landmarks*, *reference lines*, and *planes* drawn on cone-beam computed tomography images. **a** Sagittal plane – passing through the midpalatal suture, perpendicular to the horizontal and coronal planes. **b** Horizontal plane – the palatal plane. **c** Coronal plane – passing through the *right* and *left* greater palatine foramina. Abbreviations: *R* right, *L* left, *P* posterior, *A* anterior

As a part of pre-treatment examination of each patient, CBCT (Finecube; Yoshida Dental MFG. Co., Tokyo, Japan) images of the maxillary and mandibular dentoalveolar regions were acquired for diagnosis and treatment planning, using the following settings: normal mode (16.8 s, 4.10 mGy, 90 kV, and 4 mA); slice thickness, 0.147 mm; field of view (FOV), 81 × 74 mm; and voxel size, 0.146 mm. All images were acquired with the head positioned along the Frankfort horizontal plane, running parallel to the floor. Images were saved as digital imaging and communication in medicine (DICOM) files, and sagittal and horizontal views of those were extracted and evaluated using an image analysis software (ImageJ version 1.48; National Institute of Mental Health, MD, USA). Prior to measurement, the three dimensions were calibrated and the three planes (i.e., sagittal, horizontal, and coronal) defined in each image (Fig. 1).

**Fig. 2:**
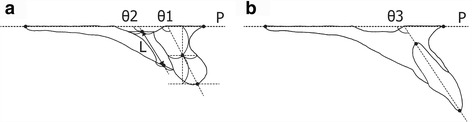
Definitions of linear and angular measurements in the midsagittal plane. **a** *PP* palatal plane; *L* length of the incisive canal; *θ1* and *θ2* angles between the palatal plane and axes of the maxillary alveolar border and incisive canal, respectively. **b** *θ3* angle between the palatal plane and axis of the maxillary left central incisor

In the midsagittal plane, linear and angular measurements were defined as follows (Fig. 2):

- P: palatal plane
- L: length of the incisive canal
- θ1, θ2, and θ3: angles between the palatal plane and axes of the maxillary alveolar border, the incisive canal, and maxillary left central incisor, respectively.

**Fig. 3:**
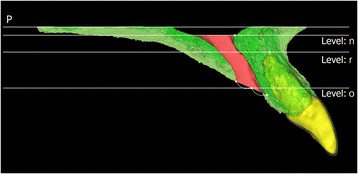
Definitions of the three vertical levels in the midsagittal plane. n, r, and o, levels of the nasal opening of the incisive canal, root apex of the maxillary incisor, and oral opening of the incisive canal, respectively. *P* palatal plane

**Fig. 4:**
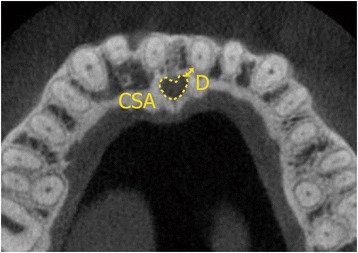
Definitions of linear and area measurements in the horizontal plane. D distance from the *left* maxillary incisor to the incisive canal (mm); *CSA*, area of the incisive canal (mm^2^)

Linear and area measurements were acquired in the horizontal plane at three vertical levels: n, r, and o (levels of the nasal opening of the incisive canal, root apex of the maxillary incisor, and oral opening of the incisive canal, respectively; Fig. 3), and the distance from maxillary incisors to the incisive canal (D) and the cross-sectional area of the incisive canal (CSA) were measured at each level (Dn, Dr, and Do, respectively; CSAn, CSAr, and CSAo, respectively; Fig. 4). All measurements were performed by a single examiner, who repeated each measurement after a 2-month interval. The Dahlberg formula was used to calculate method error, as follows:

$$ \mathrm{S}\mathrm{e} = \sqrt{\left({\displaystyle {\sum}_{i=1}^n\frac{d_i^2}{2n}}\right)} $$

where *d* = difference between two measurements and *n* = number of measurement pairs [17].

### Statistical analysis

All statistical analyses were performed using the computer software package SPSS 22.0 (IBM, Armonk, NY, USA). Mean values and standard deviations (SDs) were calculated for all measurements. Comparison of variables between male and female patients was performed with the two-sample *t* test. Correlations between parameters were examined by Pearson’s correlation analysis and Bonferroni correction for multiple comparisons. The significance level for all analyses was set at *p* < 0.05.

## Results

Method errors for linear measurements L, Dr, and Do ranged between −0.2 and 0.2 mm, −0.4 and 0.5 mm, and −0.3 and 0.5 mm, respectively, while those for angular measurements θ1, θ2, and θ3 ranged between −1.5° and 2.5°, −1.4° and 1.3°, and −1.3° and 1.1°, respectively. Method errors for area measurements CSAn, CSAr, and CSAo ranged between −5.9 and 6.0 mm^2^, −5.5 and 6.2 mm^2^, and −4.9 and 5.5 mm^2^, respectively. There were no significant differences between the original and repeat measurements of any of the parameters.

**Fig. 5:**
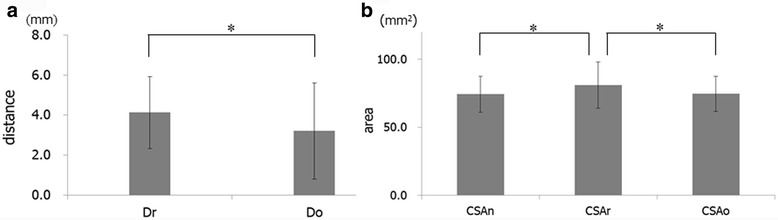
**a** Statistical comparison of the distance from the left maxillary incisor to the incisive canal at level-r (Dr) and level-o (Do) (mm). **b** Statistical comparison of the area of the incisive canal at level-n (CSAn), level-r (CSAr), and level-o (CSAo) (mm^2^). *p < 0.05

The descriptive statistics of all measurements in the sagittal plane are presented in Table 1. Only L and θ2 exhibited significant sex-specific differences. The value of L in male patients (13.8 ± 2.2 mm) was significantly greater compared to that in female patients (12.2 ± 2.3 mm; *p* < 0.05), while θ2 in male patients (105.5° ± 8.6°) was significantly smaller compared to that in female patients (109.6° ± 7.9°; *p* < 0.05). There were significant positive correlations between θ1 and θ2 (*p* < 0.01; *r* = 0.719), θ2 and θ3 (*p* < 0.01; *r* = 0.488), and θ3 and θ1 (*p* < 0.01; *r* = 0.628; Table 2). While Dr (4.1 ± 1.8 mm) was significantly greater than Do (3.2 ± 1.3 mm; *p* < 0.05), CSAr (81.0 ± 17.3 mm^2^) was significantly greater than CSAn (74.0 ± 13.5 mm^2^) and CSAo (74.5 ± 13.3 mm^2^; both *p* < 0.05; Fig. 5).

**Table 1:** Differences in linear, angular, and area measurements according to sex

Parameters	Male (*n* = 31)	Female (*n* = 62)	*p*	Gender comparison
L (mm)	13.8 ± 2.2	12.2 ± 2.3	<0.01	*
θ1 (degrees)	108.4 ± 7.7	108.4 ± 6.8	0.11	NS
θ2 (degrees)	105.5 ± 8.6	109.6 ± 7.9	0.03	*
θ3 (degrees)	112.1 ± 8.7	112.2 ± 10.3	0.98	NS
D_r_ (mm)	4.5 ± 2.4	4.0 ± 1.5	0.22	NS
D_o_ (mm)	3.1 ± 1.4	3.3 ± 1.2	0.50	NS
CSA_n_ (mm^2^)	73.9 ± 10.7	73.8 ± 14.8	0.98	NS
CSA_r_ (mm^2^)	81.4 ± 14.5	81.4 ± 18.5	>0.99	NS
CSA_o_ (mm^2^)	74.2 ± 9.3	75.0 ± 14.7	0.78	NS

Data are presented as mean ± standard deviation

*p < 0.05

*NS* not significant

**Table 2:** Statistical analysis of angular measurements θ1, θ2, and θ3

Comparisons	Correlation coefficient	*p*
θ1 vs θ2	0.719	<0.01
θ2 vs θ3	0.488	<0.01
θ3 vs θ1	0.628	<0.01

## Discussion

To our knowledge, this is the first study involving a large population to investigate the anatomical characteristics of maxillary incisors, the incisive canal, and the maxillary alveolar border using CBCT images. First, in the present study, the length of the incisive canal in male patients was significantly greater compared to that in female patients, which is concordant with the findings of a previous study [18]. Second, inclination of maxillary incisors was significantly correlated with those of the maxillary alveolar border and axis of the incisive canal. Moreover, maxillary incisors were located closer to the incisive canal at the level of the root apex than at the level of oral opening of the incisive canal, and the CSA of the incisive canal at the level of the maxillary incisor root apex was significantly greater compared to those at the levels of oral and nasal openings.

Since DICOM data are divided into 512 × 512 matrices, a large FOV corresponds to large voxel size and reduction in image resolution. In contrast, a limited FOV provides high visibility, but leads to an increase in radiation dose per unit of tissue. Thus, FOV should be set with consideration of object size for evaluation [19, 20]. In orthodontics, accurate diagnosis requires careful observation of not only of the alveolar bone but also dental roots. The FOV of CBCT images in the present study (i.e., 81 × 74 mm) was smaller in comparison with those reported in previous studies [19, 20]. This limited FOV, set in order to achieve an adequate voxel size (0.146 mm) for accurate diagnosis, provided more accurate information.

The incisive canal, an anatomical structure located on the midsagittal plane of the maxillary bone running parallel and posterior to maxillary incisors, involves the nasopalatine vessels and nerves, branches of the trigeminal nerve, and the maxillary artery and is surrounded by a thick layer of cortical bone [21–23]. Because of its proximity to maxillary incisors, the possibility of sensory dysfunction in the anterior region and failure of osseointegration has been reported in cases of contact of the incisive canal through surgical interventions such as dental implant placement [24–26]. Recently, Chung et al. reported that proximity of maxillary incisal roots to the incisive canal might influence the degree of root resorption after large incisal retraction [27]. Therefore, when planning orthodontic treatment, it is critical to confirm the exact location of maxillary incisors and the incisive canal and determine the morphology of the alveolar bone. Imaging of anatomical structures at the maxillary anterior region by CBCT with a limited FOV has not been documented in literature. In the present study, the morphologies of and positional relationships between the incisive canal and maxillary incisors were evaluated by CBCT images acquired with a limited FOV.

For calculating the degree of maxillary incisor movement, 2D analysis can provide only limited information regarding the 3D maxillofacial structures. Apical root resorption has been frequently reported in the maxillary incisor region [28]. In some cases, unexpected apical root resorption in maxillary incisors after anterior retraction has been reported to have occurred because of proximity or contact of the roots with the labial, palatal, or incisive canal cortical plates [29, 30]. In clinical settings, several orthodontic treatments till date have been successfully performed solely with conventional cephalometric analysis. However, recent temporary anchorage devices (i.e., miniscrew implants) have expanded the range of orthodontic treatment and made it possible to achieve a large degree of maxillary incisor movement [6, 27, 31]. To manage post-orthodontic treatment complications such as root resorption, gingival recession, dehiscence, and fenestration following root deviation from the alveolar bone housing, anatomical features of the maxillofacial area should be carefully examined in each patient, and diagnosis should be formulated based on 3D information. The present findings suggest that FOV-limited CBCT is a useful modality for orthodontic diagnosis of maxillary protrusion.

Loss of a maxillary incisor affects the morphology of the maxillary alveolar border and, consequently, alters the morphology of the anterior wall of the incisive canal [32]. Moreover, changes in location and inclination of the maxillary incisors lead to morphological changes in the maxillary alveolar border [12, 33–36]. Therefore, pre and post-orthodontic treatment FOV-limited CBCT analyses for assessment of morphological changes in the maxillary anterior region and the incisive canal are required to ensure precise evaluation of tooth movement-induced anatomical changes in the surrounding tissues [27, 36].

## Conclusions

Anatomic variations that are present in the anterior region of the maxillary alveolar bone yielded morphometric data that might be useful for orthodontic treatment planning in patients requiring significant correction of maxillary incisal inclination or root position or in patients requiring implant placement in the anterior region.

## Abbreviations

2D: Two-dimensional
3D: Three-dimensional
CBCT: Cone-beam computed tomography
CSA: Cross-sectional area of the incisive canal
D: The distance from the left maxillary incisor to the incisive canal
DICOM: Digital imaging and communication in medicine
FOV: Field of view
L: The length of the incisive canal
n: The level of the nasal opening of the incisive canal
o: The level of the oral opening of the incisive canal
P: Palatal plane
r: The level of the root apex of the maxillary incisor
θ1: The angles between the palatal plane and the maxillary alveolar border
θ2: The angles between the palatal plane and the incisive canal
θ3: The angles between the palatal plane and maxillary incisor
